# Supplementary figures and images for: Research on equipment fault diagnosis model based on gan and inverse PINN: Solutions for data imbalance and rare faults
Source: PLoS One. 2025 May 27;20(5):e0324180. doi: 10.1371/journal.pone.0324180 (PMC12111269; doi:10.1371/journal.pone.0324180)

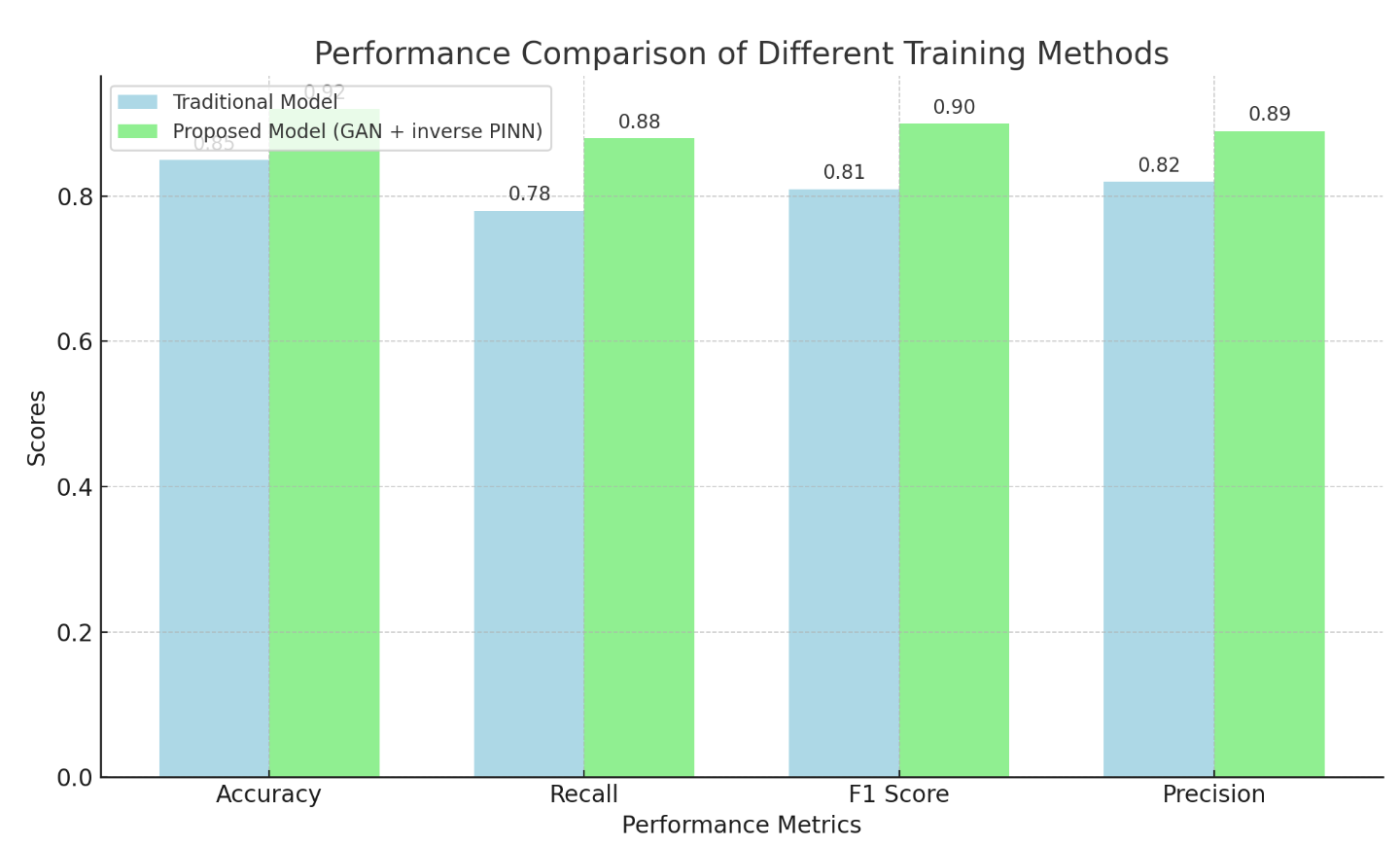

Supplement: S1 Fig — From the Figures, the Accuracy of the Inverse PINN Model Reached 94.5%, Which Is at Least a 5 Percentage Point Improvement Over Traditional Methods. At the Same Time, Recall and F1 Score Also Showed Significant Improvements, Indicating That the Inverse PINN Model Performs More Stably in Capturing Equipment Faults.The above two comparison figures illustrate the model performance using different training methods: Traditional Models vs. Suggested Model (Without Using GAN): This chart compares the performance of traditional fault detection methods with the inverse PINN model that does not use data generated by GAN. The assessed metrics include: Accuracy: The correctness of the model’s predictions, indicating the model’s ability to recognize all samples. Recall: The model’s ability to identify faults, measuring the capture rate of actual fault samples. F1 Score: The harmonic mean of accuracy and recall, providing a comprehensive assessment of the model’s performance in fault detection. Precision: The proportion of correctly classified fault samples, reflecting the reliability of the model’s predictions. (TIF) [file pone.0324180.s001.tif]

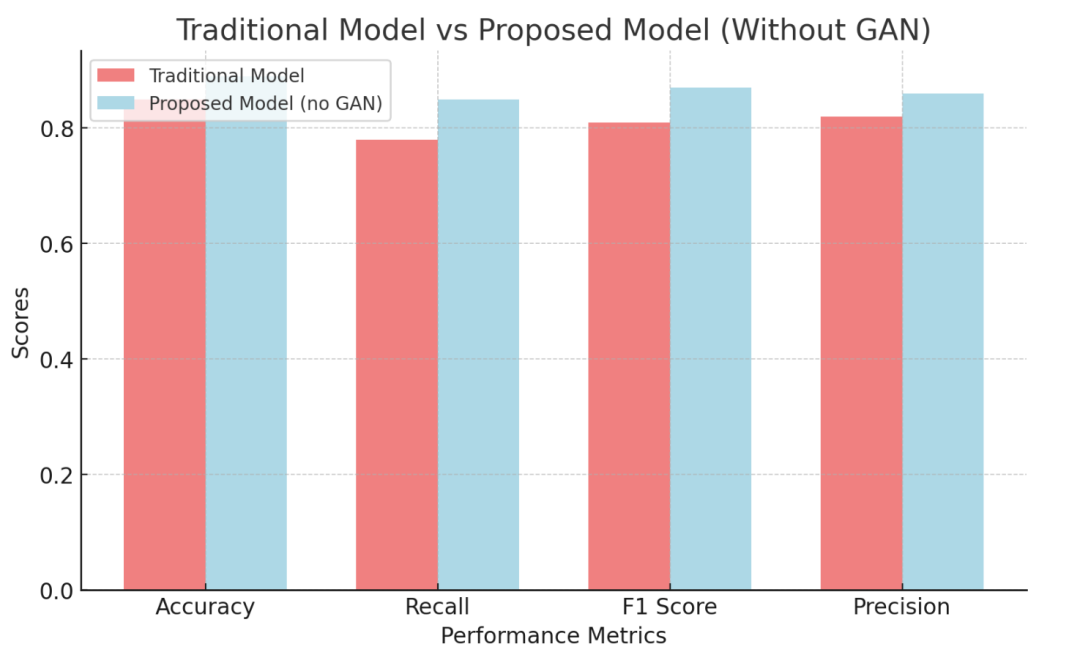

Supplement: S2 Fig — From the S2 Fig, it is evident that the suggested model (without using GAN) shows significant improvements across all metrics compared to traditional models, particularly in recall and F1 score. The traditional models exhibited lower accuracy when faced with complex faults due to a lack of data diversity and physical constraints. In contrast, the suggested model effectively enhances fault recognition capability by introducing the inverse PINN mechanism, leveraging the combination of physical models and monitoring data. Suggested Model with and without GAN-Generated Data: This chart presents the comparison results between the same model using data generated by GAN and one that does not. Key metrics include: Accuracy: After using GAN-generated data, the model’s accuracy significantly improved, indicating its enhanced ability to distinguish between normal and fault samples. Recall: By incorporating diverse fault data generated by GAN, the model is able to capture more fault instances, leading to a significant increase in recall. F1 Score: The simultaneous improvement in accuracy and recall results in a notable increase in the F1 score, demonstrating a more balanced performance of the model in fault detection. Precision: Following the inclusion of GAN data, the model’s precision improved, indicating increased confidence in identifying faults and reducing false positives. (TIF) [file pone.0324180.s002.tif]

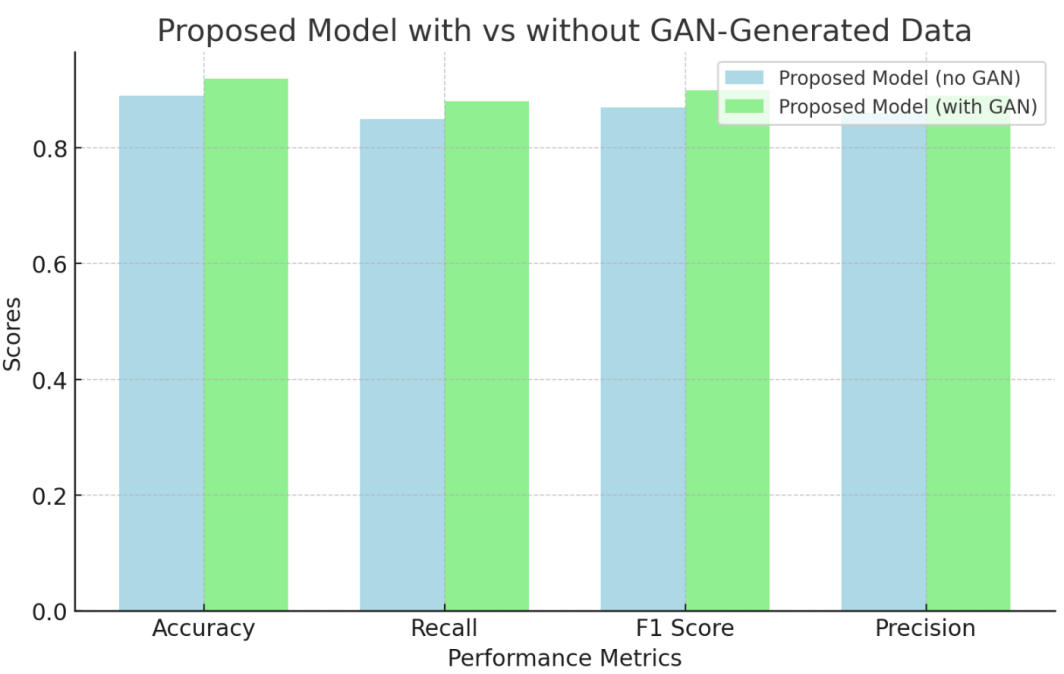

Supplement: S3 Fig — These comparison S3 Fig charts clearly demonstrate the positive impact of GAN-generated data on model performance. Through these figures, it can be intuitively observed that GAN not only effectively supplements the data and enhances the model’s robustness but also improves the overall performance of the model in fault detection.The experimental results indicate that the proposed model surpasses traditional fault diagnosis methods in all performance metrics, particularly in key indicators such as response rate and F1 score, fully proving the advantages of combining GAN-generated data with inverse PINN. (TIF) [file pone.0324180.s003.tif]
