# Supplementary material for: Research on equipment fault diagnosis model based on gan and inverse PINN: Solutions for data imbalance and rare faults
Source: PLoS One. 2025 May 27;20(5):e0324180. doi: 10.1371/journal.pone.0324180 (PMC12111269; doi:10.1371/journal.pone.0324180)
Supplement: Attachment — Submitted filename: Dear Canales.docx [file pone.0324180.s004.docx]

Dear Canales,

Thank you for your review and feedback. We are pleased to address each of the points outlined in your message.

****English Translation** of Title and Abstract**: We have now resubmitted the manuscript with all sections, including the title and abstract, fully translated into English, in accordance with the journal guidelines.

****Ethics Approval**:** Our study focuses exclusively on machine equipment diagnostics and simulation data and does not involve human subjects or human ethics considerations. There were no human subjects or data from human participants included in the research, thus no human ethics approval was necessary. The equipment data was obtained with full authorization from the hospital

****Data Access Dates**:** The operational data used in this study was collected on June 2024, and this information has been added to the Methods section of our manuscript.

****Ethics Statement and Consent**:** As this research pertains solely to machinery diagnostics and simulation data, there was no need for human consent, as no human participants were involved. The equipment data was obtained with explicit approval from the hospital administration, with oversight by the third author, who serves as the hospital director.

Please let us know if any furture

Best regards,
Jackie Cheng
