# Supplementary material for: Research on equipment fault diagnosis model based on gan and inverse PINN: Solutions for data imbalance and rare faults
Source: PLoS One. 2025 May 27;20(5):e0324180. doi: 10.1371/journal.pone.0324180 (PMC12111269; doi:10.1371/journal.pone.0324180)
Supplement: Attachment — Submitted filename: Response to Reviewers.docx [file pone.0324180.s005.docx]

## ****Response to Reviewers****

We sincerely appreciate the constructive feedback provided by the reviewers. Below, we address each comment in detail and highlight the revisions made to the manuscript accordingly.

## ****Response to Reviewer #1****

**1) Emphasizing the practical value of this research in the abstract**

**Response:**
We have revised the abstract to explicitly highlight the practical significance of our research. The modified abstract now clearly states how the proposed **digital twin and inverse PINN-based fault diagnosis model** enhances **medical imaging equipment reliability**, reduces **maintenance costs**, and improves **fault prediction accuracy**. This revision ensures that the **engineering background and practical relevance** of our study are well-articulated to the readers.

**2) Summarizing research gaps before introducing contributions and discussing emerging ML applications in industrial settings**

**Response:**
We have enhanced the introduction by summarizing the key **research gaps** before presenting the **contributions and novelty** of our work. Specifically, we have identified the following gaps:

- **Limited integration of physics-based constraints with deep learning models in medical imaging fault diagnosis**
- **Challenges in addressing data imbalance in fault detection for complex equipment**
- **Lack of real-time adaptability in existing diagnostic models**

Additionally, we have expanded the discussion on **emerging ML applications in industrial settings**, particularly in:

- **Digital twin-driven intelligent assessment of gear surface degradation**
- **Cyclostationary-based straight bevel gear wear monitoring in intelligent manufacturing**
- **Cross-modal zero-shot fault diagnosis using multi-source heterogeneous non-contact sensing data**

These additions provide a broader perspective on how digital twin technology and deep learning can be applied beyond **medical imaging equipment fault diagnosis**, making our work more comprehensive.

**3) Improving the resolution of figures**

**Response:**
We have updated all figures in the manuscript to ensure **higher resolution and better clarity**. The figures are now presented in **300 DPI resolution** to improve readability in the final publication format.

**4) Addressing grammatical errors**

**Response:**
We have conducted a thorough **proofreading and grammar check** of the entire manuscript. The revised version has eliminated grammatical errors, improved sentence structures, and enhanced readability.

**5) Adding future work suggestions in the conclusion**

**Response:**
We have expanded the conclusion section to include **future research directions**, which focus on:

- **Enhancing the scalability of the proposed model** to handle **large-scale medical imaging systems** and complex industrial equipment
- **Investigating hybrid deep learning models** that integrate **transformers and graph neural networks** for fault diagnosis
- **Developing real-time digital twin-based adaptive fault detection systems** with **edge computing integration**

This addition provides a clear roadmap for extending our research.

## ****Response to Reviewer #2****

**1) Clarifying the specific medical imaging equipment used in the experiments**

**Response:**
We have now explicitly stated in the methodology section that the **United Imaging uCT760 CT scanner** was used as the testbed for our experiments. The description includes:

- **Key specifications of the uCT760 scanner**
- **The types of sensor data collected (temperature, pressure, vibration)**
- **How the data was utilized to train and validate the inverse PINN model**

This revision ensures **transparency and reproducibility** in our experimental setup.

**2) Providing more details on the implementation of digital twin components**

**Response:**
We have added an in-depth explanation of the **digital twin framework**, detailing:

- **The architecture, data flow, and synchronization mechanisms**
- **How real-time sensor data is integrated with the physics-based simulation models**
- **How the digital twin dynamically updates based on real-time fault detection results**

These improvements make the **implementation details of the digital twin model more explicit**.

**3) Explaining the relationship between physical constraints and neural network architecture**

**Response:**
We have now clearly articulated how **physical constraints are embedded into the inverse PINN model**:

- **Physics-based loss functions** are incorporated to ensure compliance with heat conduction and vibration propagation equations
- **Multi-objective optimization** balances **data-driven learning** and **physical consistency**
- **The mathematical formulation** of the inverse PINN is now explicitly provided, illustrating how **temperature, pressure, and vibration constraints guide network training**

These refinements clarify the interaction between **physics-based constraints** and **deep learning components** in our model.

**4) Comparing our approach with other state-of-the-art hybrid deep learning models**

**Response:**
We have now expanded the **related work and experimental comparison sections** to include:

- **Performance benchmarking against alternative hybrid fault diagnosis models** (e.g., transformer-based fault detection, GAN-enhanced fault classification)
- **A comprehensive table comparing accuracy, recall, and F1-score of different models**
- **Discussion on how the integration of inverse PINN with GAN-based fault augmentation improves generalization performance**

These additions strengthen the validity of our approach by positioning it within the **broader landscape of advanced deep learning-based fault diagnosis techniques**.

### ****Final Summary of Revisions****

| ****Reviewer Comment**** | ****Response and Revision**** |
| --- | --- |
| **Emphasizing the practical value in the abstract** | Added explicit discussion on how our model improves medical imaging equipment reliability and reduces maintenance costs. |
| **Summarizing research gaps before introducing contributions** | Clearly outlined research gaps before presenting the novelty of our approach. |
| **Discussing emerging ML applications in industrial settings** | Expanded discussion on digital twin-driven gear degradation assessment, cyclostationary-based monitoring, and cross-modal zero-shot diagnosis. |
| **Improving figure resolution** | Updated all figures to high-resolution 300 DPI format. |
| **Addressing grammatical errors** | Conducted thorough proofreading and improved manuscript readability. |
| **Adding future work suggestions in the conclusion** | Included discussions on scalability, hybrid models, and real-time adaptation. |
| **Clarifying the medical imaging equipment used** | Stated that the United Imaging uCT760 CT scanner was used and detailed its data collection process. |
| **Providing more digital twin implementation details** | Added descriptions of digital twin architecture, real-time data flow, and physics-based simulation integration. |
| **Clarifying physical constraints in the neural network** | Explicitly formulated how physics-based constraints are embedded in the inverse PINN model. |
| **Comparing with other hybrid deep learning models** | Conducted a comprehensive comparison with state-of-the-art hybrid models and provided benchmarking results. |

These revisions significantly **enhance the clarity, depth, and completeness** of our manuscript. We appreciate the reviewers' insightful comments, which have helped improve the quality and impact of our work.

We look forward to further feedback and are happy to make additional refinements as needed.

**Thank you for your time and consideration.**
