# Supplementary material for: Research on equipment fault diagnosis model based on gan and inverse PINN: Solutions for data imbalance and rare faults
Source: PLoS One. 2025 May 27;20(5):e0324180. doi: 10.1371/journal.pone.0324180 (PMC12111269; doi:10.1371/journal.pone.0324180)
Supplement: Attachment — Submitted filename: Response_to_Reviewers_auresp_2.docx [file pone.0324180.s006.docx]

****Response to Reviewers****

Dear Reviewers,

Thank you for your thorough and constructive feedback on our manuscript. We have carefully addressed each of your concerns and revised the paper accordingly. Below is our point-by-point response to your queries:

### ****1. Structural and Stylistic Consistency****

****Revisions****:

- We restructured the paper to ensure logical flow, particularly in Sections B–D, by introducing clearer transitions between technical concepts (e.g., adding Section D to explicitly link GAN and Inverse PINN).
- The language has been standardized to maintain formal academic tone throughout, with casual phrasing removed and technical terminology consistently applied.
- Key terms (e.g., "Digital Twin," "Inverse PINN") are now defined in a dedicated glossary section.

### ****2. Theoretical Foundation of GAN and Inverse PINN****

****Revisions****:

- A new Section D ("Synergistic Integration of GAN and Inverse PINN") has been added to explicitly detail their complementary roles:
  i. ****Data Augmentation****: GAN expands sparse fault datasets for Inverse PINN.
  ii. ****Physics-Guided Constraints****: Inverse PINN enforces physical plausibility in GAN-generated samples.
  iii. ****Regularization****: Joint optimization improves generalization.
- Mathematical formulations (e.g., combined loss functions) are now presented .

### ****3. Experimental Hyperparameters****

****Revisions****:

- Specific hyperparameters for GAN and Inverse PINN are now detailed in Section:
  - ****GAN****: 3-layer MLPs, learning rate = 0.0002, batch size = 64, 500 epochs.
  - ****Inverse PINN****: 5-layer NN, learning rate = 0.001, batch size = 32, 1000 epochs.
- Implementation details (e.g., PyTorch code snippets) are provided for reproducibility.

### ****4. Comparison with Recent Deep-Learning Methods****

****Revisions****:

- Table has been expanded to include:
  - ****Self-Supervised Learning (SSL)****and ****Physics-Guided Transformers****.
- A dedicated discussion on limitations of hybrid transformer models, highlights their computational costs compared to our physics-informed approach.

### ****5. Limitations of GAN-Generated Data****

****Revisions****:

- A new subsection in the conclusion explicitly addresses:
  i. Synthetic data realism and GAN training instability.
  ii. Mitigation strategies (e.g., WGAN-GP, physics-based validation).
- Emphasis is placed on future work to integrate physics-based validation metrics (e.g., finite element residuals).

### ****6. Mathematical Formulations****

****Revisions****:

- All equations are now consistently formatted using LaTeX (e.g., Equation 1: Combined loss function for Inverse PINN-GAN; Equation 2: Fault Severity Index).
- A new appendix provides detailed derivations of physical constraints (e.g., heat conduction PDEs).

### ****7. Digital Twin Architecture****

****Revisions****:

- Section now includes technical specifications in detail.

### ****8. Specific Future Directions****

****Revisions****:

- Future prospects are reorganized into six actionable technical directions:
  i. Cross-Domain Model Adaptation (e.g., transfer learning for MRI/ECG).
  ii. Edge-Cloud Collaborative Learning (e.g., lightweight neural networks).
  iii. Safety-Critical Validation Metrics (e.g., ISO 13485-aligned fault indices).
- Implementation roadmaps (short/medium/long-term) are provided for translational impact.

### ****Final Note****

These revisions have significantly strengthened the paper’s theoretical rigor, reproducibility, and clinical relevance. We are grateful for your insights, which have helped us refine our contributions to the field of fault diagnosis. Please let us know if further adjustments are required.

Sincerely,
Zheng Cheng
